# Osteogenesis Performance of Boronized Ti6Al4V/HA Composites Prepared by Microwave Sintering: In Vitro and In Vivo Studies

**DOI:** 10.3390/ma15144985

**Published:** 2022-07-18

**Authors:** Zhenyu Ding, Qian Peng, Jun Zuo, Yuehong Wang, Hongbo Zhou, Zhangui Tang

**Affiliations:** 1 Xiangya Stomatological Hospital, Central South University, Changsha 410008, China; dingzhenyu1993@gmail.com (Z.D.); zuolaoshimentu@csu.edu.cn (J.Z.); wangyuehong1999@163.com (Y.W.); zhb2540@csu.edu.cn (H.Z.); zhgtang@csu.edu.cn (Z.T.); 2 Xiangya School of Stomatology, Central South University, Changsha 410008, China; 3 Hunan Key Laboratory of Oral Health Research, Central South University, Changsha 410008, China

**Keywords:** Ti6Al4V, hydroxyapatite, microwave sintering, osteogenesis performance, dental implant

## Abstract

The boronized Ti6Al4V/HA composite is deemed to be an important biomaterial because of its potential remarkable mechanical and biological properties. This paper reports the osteogenesis performance of the boronized Ti6Al4V/HA composite, which was prepared by microwave sintering of powders of Ti6Al4V, hydroxyapatite (HA), and TiB_2_ in high-purity Ar gas at 1050 °C for 30 min, as dental implant based on both cell experiments in vitro and animal experiments in vivo. The comparison between the boronized Ti6Al4V/HA composite and Ti, Ti6Al4V, and boronized Ti6Al4V in the terms of adhesion, proliferation, alkaline phosphate (ALP) activity, and mineralization of MG-63 cells on their surfaces confirmed that the composite exhibited the best inductive osteogenesis potential. It exerted a more significant effect on promoting the early osteogenic differentiation of osteoblasts and exhibited the maximum optical density (OD) value in the MTT assay and the highest levels of ALP activity and mineralization ability, primarily ascribed to its bioactive HA component, porous structure, and relatively rough micro-morphology. The in vivo study in rabbits based on the micro-computed tomography (micro-CT) analysis, histological and histomorphometric evaluation, and biomechanical testing further confirmed that the boronized Ti6Al4V/HA composite had the highest new bone formation potential and the best osseointegration property after implantation for up to 12 weeks, mainly revealed by the measured values of bone volume fraction, bone implant contact, and maximum push-out force which, for example, reached 48.64%, 61%, and 150.3 ± 6.07 N at the 12th week. Owing to these inspiring features, it can serve as a highly promising dental implant.

## 1. Introduction

Pure titanium and titanium alloys, e.g., Ti6Al4V, are widely used in dental implantation because of their good mechanical properties, biocompatibility, and stable chemical properties [1]. However, the surfaces of titanium and its alloys are bio-inert and their binding with bone lacks mechanical strength. After being implanted into the alveolar bone, they may lack primary stability and thus require long healing time to avoid repair failure [2]. Therefore, since 1960s, many efforts have been made for the fabrication of composites constituted by a metal substrate and a coating layer of bioactive substance, often hydroxyapatite (HA), which possesses great biological activity [3]. However, for these composites, they usually have poor binding strength between the substrate and coating due to obvious property differences between the metal and ceramic material.

Unlike coating and other surface modification methods, powder sintering is also commonly used for preparing titanium alloy/HA composites [4]. This method can not only realize the high-strength integration of each component of composites and then strengthen the mechanical properties, but also stabilize the physical structure and chemical composition of the easily decomposed phase to ensure and improve biological properties. It is worth noting that compared with the traditional sintering technology, the combination of external field technologies, such as clean microwave energy, has potential to strengthen the sintering process, greatly reducing the sintering temperature and time, which avoids HA decomposition with sufficient mechanical properties [5]. 

For obtaining implant materials with more excellent mechanical properties and biological activity, titanium alloy matrix and HA powder can be modified in advance with other elements. For example, boron (B) plays an important role in maintaining joint health, enhancing bone density and fracture healing in human body. With the addition of boron in the forms of TiB_2_ and other compounds to the alloy, its mechanical properties and corrosion resistance could be enhanced mainly due to the in situ formation of TiB [6]. Most recently, the authors’ group successfully prepared boronized Ti6Al4V/HA composites as dental implants by microwave sintering of powders of Ti6Al4V in the presence of 5% HA and 5% TiB_2_ [7,8] at 1050 °C for 30 min in the gas of high-purity Ar. It was revealed that the composite could obtain satisfactory comprehensive mechanical properties and good biological activity. In specific, it had compressive strength of 351.1 MPa, compressive modulus of 39.2 GPa, microhardness of 462.8 HV, failure resistance of 0.1158, and anti-wear property of 0.1325 GPa, which were superior to those of other related composites and natural bones. Moreover, by immersing this composite in the simulated body fluid (SBF), evident HA precipitation occurred on its surface, showing its great bioactivity.

The above findings showed that the boronized Ti6Al4V/HA composite is a promising dental implant material with excellent quality in terms of basic mechanical properties and bioactivity. However, to evaluate its clinical application value, its osteogenesis performance should be investigated in depth. In view of the deficiencies of relevant reports on this aspect, the purpose of the present study was to explore in vitro inductive osteogenesis potential and in vivo osseointegration behavior of the boronized Ti6Al4V/HA composite for confirming its applications to dental implantation based on its comparison with popular pure titanium, Ti6Al4V and boronized Ti6Al4V. 

## 2. Materials and Methods

### 2.1. Samples and Animals

**Figure 1:**
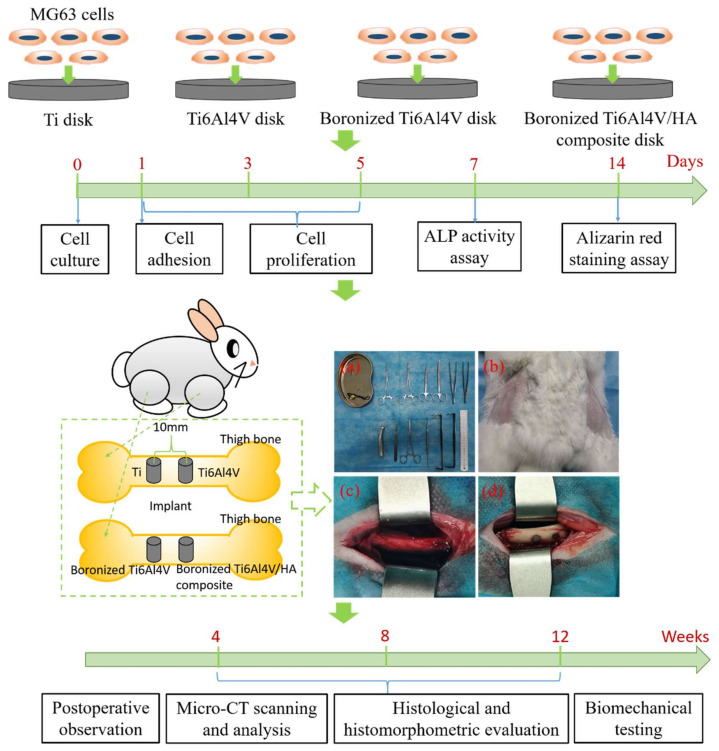
Illustration of cell experiments in vitro and animal experiments in vivo: (**a**) operation facilities, (**b**) preparation of operation skin site, (**c**) bone surface of exposed operation area, and (**d**) insertion of implants into the bone cavity of rabbit.

For cell experiments in vitro, commercial pure titanium disks (CpTi, Ø 8 mm × 2 mm) and Ti6Al4V disks (Ø 8 mm × 2 mm) were purchased from the Guanyue Metal Materials Co., Ltd. The samples, including boronized Ti6Al4V disks (raw material mass ratio: 95% Ti6Al4V (15–50 μm) + 0% HA (needle-like shape with a length of about 100 nm and a diameter of about 20 nm) + 5% TiB_2_ (4–8 μm), Ø 8 mm × 2 mm) and boronized Ti6Al4V/HA composite disks (raw material mass ratio: 90% Ti6Al4V + 5% HA + 5% TiB_2_, Ø 8 mm × 2 mm), were prepared by microwave sintering at 1050 ℃ for 30 min in the high-purity argon gas after blending and milling of the raw materials with extra addition of 3% stearic acid as binder in a high-energy ball mill at a speed of 200 r/min for 12 h with the mass ratio of stainless steel ball to the sample of 10, pressing using a hydraulic briquetting machine at pressure of 900 MPa, and debinding in a conventional tube furnace at 380 °C for 2 h with a heating rate of 3 °C/min in an inert atmosphere [7]. The main phases of the sintered composite included α/β-Ti, Ca_10_(PO_4_)_6_(OH)_2_, and TiB which acted as a transitional component for integrating titanium matrix and hydroxyapatite due to the in situ formation of TiB that bound them. More details about the physiochemical features of the composite are available in the previous studies [7,8]. All samples were polished with sand paper by 240#, 600#, 800#, 1000#, 1500#, 2000# in sequence. They were then put into acetone, anhydrous ethanol, and deionized water for ultrasonic cleaning for 15 min, respectively. After drying in a vacuum drying oven and sterilizing under high temperature and pressure conditions, they were ready for in vitro study following the steps including cell culture, cell adhesion, cell proliferation, alkaline phosphatase (ALP) activity assay, and alizarin red staining assay. For animal experiments in vivo, they were approved by the Experimental Animal Welfare and Ethics Committee of Central South University. In the experiments, 18 white New Zealand rabbits were used, with no regard to gender. They were between 3 and 4 months of age, each with a body weight of 2.5–3.0 kg. All rabbits were kept in separate cages for 1 week before surgeries. For implantation, a total of 72 implants of pure Ti (control 1), Ti6Al4V (control 2), boronized Ti6Al4V (control 3), and boronized Ti6Al4V/HA composite (experimental) with the size of Ø 3 mm × 5 mm, prepared by the same method mentioned before, were used in the experiments. They were divided into four groups (*n* = 6 at 4th, 8th, and 12th weeks of post implantation). All the implants were sterilized by ultrasonic oscillation, and packaged by sterile bags, and then autoclaved before being inserted into the rabbits. All the 72 implants were placed in the right and left sides (2 implants per side, with the distance of 10 mm)) of rabbit femurs (18 animals) and evaluated after 4, 8, and 12 weeks following the implantation surgeries (Figure 1).

### 2.2. In Vitro Study

#### 2.2.1. Cell Culture

MG-63 cells were maintained in α-minimum essential medium (MEM) (Gibco, Invitrogen, NY, USA) containing 10% FBS (Gibco, Invitrogen, NY, USA), 100 U/mL penicillin (Gibco, Invitrogen, NY, USA), and 100 U/mL streptomycin (Gibco, Invitrogen, NY, USA). The cells were cultured under 100% humidity and 5% CO_2_ at 37 °C. The medium was changed every 3 days and after the confluence, the cells were digested with 0.25% trypsin/0.02% EDTA. The cells from passage 2 to 4 were used for the experiments because they had the best cell viability.

#### 2.2.2. Cell Adhesion

The cell adhesion assay was used to investigate the morphology and focal adhesion development in adherent MG-63 cells on the investigated samples at 24 h of culture using scanning electron microscopy (SEM). The cells were cultured on Ti disks, Ti6Al4V disks, boronized Ti6Al4V disks, and boronized Ti6Al4V/HA composite disks at an initial seeding density of 1 × 10^4^ cells/well, respectively. For SEM observation, the MG-63 cells spreading on the investigated samples were fixed with 4% paraformaldehyde and then dehydrated using an ascending series of alcohols. After drying and gold-palladium coating, the cell morphologies were characterized. 

#### 2.2.3. Cell Proliferation

The MG-63 cells were cultured on Ti disks, Ti6Al4V disks, boronized Ti6Al4V disks, and boronized Ti6Al4V/HA composite disks in 24-well culture plates at an initial seeding density of 2 × 10^4^ cells/well for evaluation of cell proliferation. They were cultured for 1, 3, and 5 days for cellular proliferation assay according to the 3-(4,5-dimethylthiazol-2-yl)-2,5-diphenyltetrazolium bromide (MTT) method in accordance with the manufacturer’s instructions (*n* = 9 per group). The values of optical density (OD) for the samples were determined based on the absorbance measurements at 490 nm.

#### 2.2.4. ALP Activity Assay

The ALP activity is a sign of early osteogenic differentiation of MG-63 cells. The total cellular ALP activity in the cell lysates was measured in 2-amino-2-methyl-1-propanol buffer with p-nitrophenyl phosphate as the substrate at 7 days of culture at an initial seeding density of 2 × 10^4^ cells/well. The absorbance change at 405 nm was measured using a microplate reader (*n* = 6 per group). Total protein was extracted from the cell lysates and quantified with a BCA Protein Assay kit (Beyotime Biotech, Haimen, China). The ALP activity was expressed as nanomoles of p-nitrophenol liberated per microgram of total cellular protein.

#### 2.2.5. Alizarin Red Staining Assay

Mineralized nodule is a sign of osteogenic differentiation and maturation of MG-63 cells. Alizarin red can be chelated with calcium ions to produce a deep red or purplish red complex. The mineralization analyses were conducted by alizarin red staining after 14 days of culture at an initial seeding density of 2 × 10^4^ cells/well (*n* = 3 per group) and the mineralization percentage was determined by a microplate reader at 405 nm absorbance (*n* = 3 per group). 

### 2.3. In Vivo Study

#### 2.3.1. Surgical Procedures

All animals were operated under general anesthesia during the surgery. General anesthesia was induced by xylazine hydrochloride at a rate of 0.03 mL/kg, with an intramuscular injection. The surgical sites of animals were shaved and sterilized by iodine before they were incised with a scalpel. The medial aspects of the proximal femoral metaphysis were then exposed since skin, muscles, and periosteal layers were pulled away from the surgical sites. Two implant cavities were prepared in each femoral metaphysis (proximally and distally) using a precision drill and a tapered drill (Nobel Biocare AB, Box 5190, Goteborg, Sweden) at a rotation speed of 1000 rpm. The implant cavities of 3 mm in diameter and 5 mm in depth were formed perpendicular to the long axes of the left and right femoral cortical bones. The implants were subsequently installed into the left and right femurs of the animals, respectively. The subcutaneous layer and skin of the wound were separately sutured with 4-0 nylon sutures after the surgery. The animals were housed in cages after the surgery and received an intramuscular injection of penicillin at a dose of 0.8 million units/day for 3 days to prevent postsurgical infection. At 4, 8, and 12 weeks of healing, the rabbits were euthanized under general anesthesia. The femurs containing implants and bone were cut out and stored in formalin as specimens for further evaluation.

#### 2.3.2. Postoperative Observation

After the surgeries, the rabbits were examined systematically, including feeding, activity, wound healing, infection, and pain.

#### 2.3.3. Micro-CT Scanning and Analysis 

The specimens were scanned with a resolution of 19 × 19 × 19 μm. A micro-CT analysis software (SIEMENS, Munich, Germany) was used to reconstruct 3-D images of the specimens. A cylindrical area was selected as the region of interest (ROI) with the width designed as 0.5 mm outside of implant margin and the length designed as 0.5 mm in the along axis of the implant. The trabecular volume (TV), bone volume (BV), bone volume fraction (BV/TV), trabecular number (Tb.N), trabecular thickness (Tb.Th), trabecular separation (Tb.Sp), bone surface (BS), BS/BV, BS/TV, and bone mineral density (BMD) were determined based on the micro-CT 3D reconstructions.

#### 2.3.4. Histological and Histomorphometric Evaluation

The specimens stored in formalin were washed and dehydrated using an ascending series of alcohols and embedded in methyl methacrylate for undecalcified sectioning. Undecalcified cut-and-ground sections, which were prepared in a plane parallel to the long axis of each implant and contained the central part of the implants, were produced at a final thickness of 200 μm using a cutting and grinding system (EXAKT E300CP/400CS, Germany). The sections were stained with toluidine blue and the histomorphometric analysis was carried out using light microscopy (DP72, Olympus, Tokyo, Japan) with an image analysis system. The images were captured using a digital camera attached to the microscope and displayed on a computer monitor. The bone implant contact (BIC), which was determined by the ratio of the length of mineralized bone in direct contact with the implant to the total lateral length of the implant, was then measured.

#### 2.3.5. Biomechanical Testing

The bone-implant interfacial strength was determined at 4, 8, and 12 weeks by pullout tests. The paired femurs were dissected from the limbs immediately after euthanasia, stripping of soft tissues, wrapping in PBS-soaked gauze, and storage at 4 °C. The implants were tested within 24 h of collection. The femur was mounted in polymethylmethacrylate blocks and connected to a servohydraulic testing machine (Instron, Canton, MA, USA) by a push-off pin. A pullout-force vector was applied to the long axis of the implant at a constant displacement rate of 0.5 mm/min and the force-displacement curve was recorded. The peak load (maximum push out force, Fmax) before failure was divided by the exterior surface area of the implant to determine the interfacial strength.

## 3. Results and Discussion

### 3.1. Cell Adhesion Behavior

**Figure 2:**
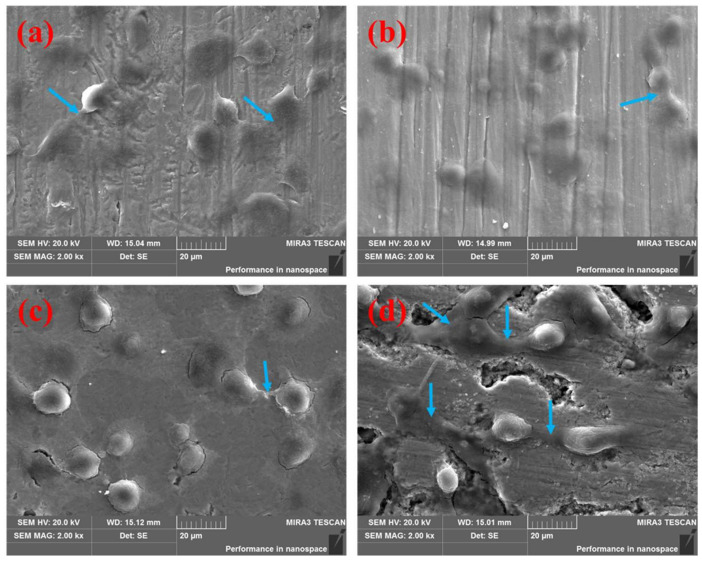
SEM images of surfaces of the different specimens after cell culture for 12 h with the blue arrows showing the pseudopodia between cells: (**a**) Ti, (**b**) Ti6Al4V, (**c**) boronized Ti6Al4V, and (**d**) boronized Ti6Al4V/HA composite.

After cell cultivation for 12 h, the adhesion of cells on the surfaces of the four different groups of specimens was observed, as shown in Figure 2.

Figure 2 shows that the cells could adhere and migrate on the surfaces of all four groups of implants. The cells on the surfaces of Ti, Ti6Al4V, and the boronized Ti6Al4V/HA composite were oblate, fusiform, and polygonous. Multiple pseudopodia, highlighted by the blue arrows in Figure 2, were cross-linked with each other on the surfaces of these implants, indicating good adhesion of the cells. Longer and more closely connected pseudopodia were observed on the surface of boronized Ti6Al4V/HA composite compared with Ti and Ti6Al4V. It showed higher degrees of cell adhesion, growth, and differentiation of the composite. On the other hand, there were spherical cells with less pseudopodia on the surface of boronized Ti6Al4V, indicating its limited cell adhesion. This phenomenon was attributed to the implant–cell interaction. It is known that the van der Waals force and intermolecular attraction enable cells to adhere on the materials, depending on the various ingredients, structure, and morphology of material surface [9]. The integration between specimen surfaces and proteins could change the cell morphology, making cells become fusiform and polygonal to adhere on the surfaces of material tightly. In the sintering process, the interfaces between different phase components were formed in the composite [7], which could help the cells adhere on the material surface. Meanwhile, many micro-pores were formed during the process of sintering, promoting adhesion and proliferation of cells [10]. In addition, HA in the composite implant could exchange ions with cell culture medium. As a result, the surface charge was changed, producing highly concentrated calcium and phosphorous elements which absorbed proteins and improved cellular chemotaxis and adhesion [11,12]. Compared with other implants, the Ti6Al4V/HA composite possessed advantages in terms of microstructure and composition. Therefore, it exhibited the best capability to promote cellular adhesion. 

### 3.2. Cell Proliferation Behavior

**Figure 3:**
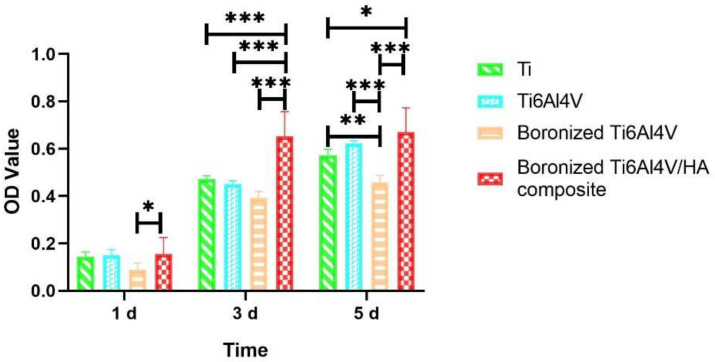
Cell proliferations on the different specimens after 1, 3, and 5 d of incubation determined by the MTT assays (* *p* < 0.05, ** *p* < 0.01, and *** *p* < 0.001).

Figure 3 shows the cell proliferations of the different specimens determined by the MTT assays on 1 d, 3 d, and 5 d. 

According to Figure 3, on day 1, the MG-63 cells on all specimens started to proliferate and there was no obvious difference between the groups, except for boronized Ti6Al4V. It was worse than the other groups with a significant difference between boronized Ti6Al4V and boronized Ti6Al4V/HA composite (*p* < 0.05), probably related to the uneven distribution of boride when the boronized alloy was prepared without the participation of nanoscale HA. On day 3, there were increased proliferations of cells in all groups. Especially, the peak value of boronized Ti6Al4V/HA composite was higher than those of the other groups, with a significant difference (*p* < 0.001). The proliferation activities of Ti, Ti6Al4V and boronized Ti6Al4V increased compared with the counterparts on day 1, but there was no statistical difference between them (*p* > 0.05). On day 5, the speed of proliferation slowed and reached a plateau. However, there were significant differences between the groups and the boronized Ti6Al4V/HA composite which showed the highest ability to proliferate. On the other hand, the MG-63 cells had the slowest proliferative speed on the boronized Ti6Al4V, followed by Ti and Ti6Al4V. It indicated that the addition of B may partially restrain the cellular proliferation and differentiation although it is a nutrient element. A systematic study of the effect of addition of boron is ongoing. Conversely, HA which was located between the alloy grains of boronized Ti6Al4V/HA composite, as observed in the previous study [7], could resolve and release calcium ions to promote proliferation and differentiation of osteogenic cells [13,14,15]. It would integrate with integrin in cellular surface (such as β1-integrin) and matrix protein (such as fibronectin and I type collagen) and trigger inner signal transduction, thus promoting cellular proliferation and differentiation and eventually exhibiting superior proliferative viability [16,17]. 

### 3.3. ALP Activity Analysis

**Figure 4:**
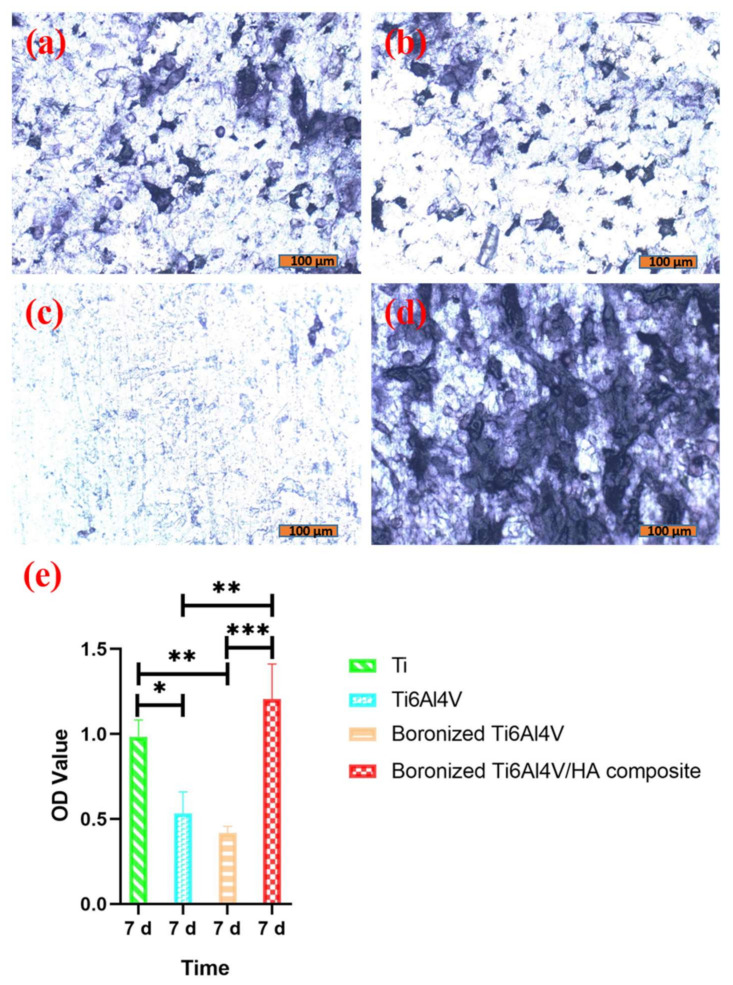
ALP staining of cells cultured on the different specimens for 7 days: (**a**) Ti, (**b**) Ti6Al4V, (**c**) boronized Ti6Al4V, and (**d**) boronized Ti6Al4V/HA composite. (**e**) ALP activity of cells cultured on the different specimens for 7 days (* *p* < 0.05, ** *p* < 0.01, and *** *p* < 0.001).

The cell differentiation abilities on the different specimens were determined based on analysis of the ALP activity of cells because ALP is a marker of the early stage of osteogenic differentiation [18]. The results are shown in Figure 4.

As shown in Figure 4, the cells on the surfaces of Ti and Ti6Al4V were well differentiated, as indicated by many purple black cells. On the contrary, the cells on the surface of boronized Ti6Al4V showed a low differentiation level, as demonstrated by only a few scattered stained cells. The best cell differentiation was observed on the surface of boronized Ti6Al4V/HA composite. There were many tightly connected dark purple cells with purple particles in the cytoplasm. 

According to the ALP activity determination in Figure 4e, the cells on the boronized Ti6Al4V/HA composite possessed the highest ALP activity. Despite no statistical difference from commercial pure Ti, there were significant differences when compared with Ti6Al4V and boronized Ti6Al4V. Obviously, the boronized Ti6Al4V/HA composite could facilitate osteoblast differentiation. It could be ascribed to its special Ca-containing surface composition, porous structure, and relatively rough micro-morphology, as well as the wnt/β-catenin and BMP/SMAD signal transduction system activated by integrin and matrix protein in the cells [19,20]. 

### 3.4. Alizarin Red Staining Analysis

**Figure 5:**
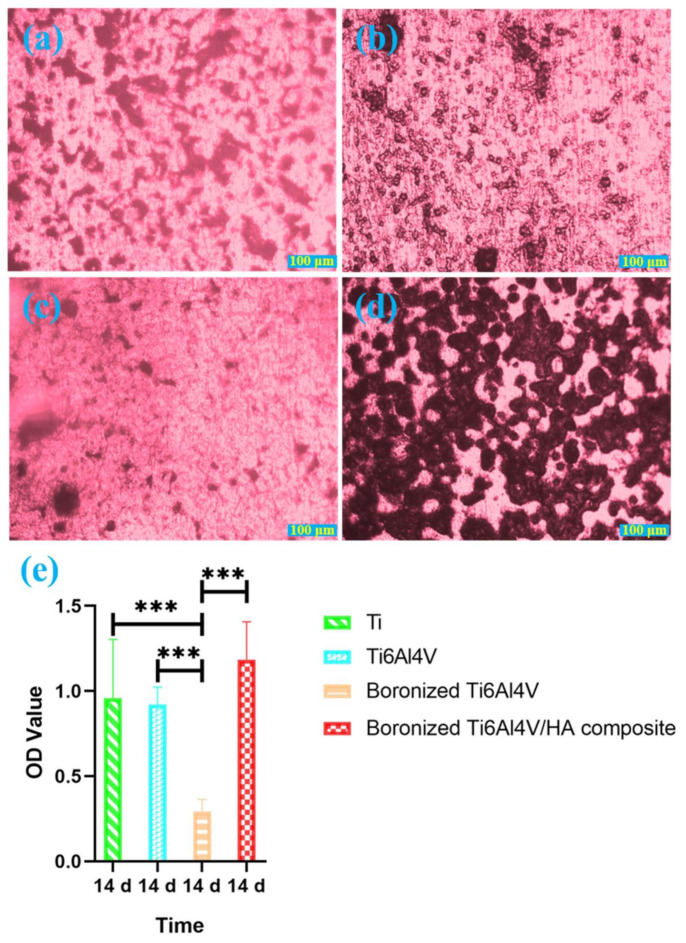
Alizarin red staining of cells cultured on the different specimens for 14 days: (**a**) Ti, (**b**) Ti6Al4V, (**c**) boronized Ti6Al4V, (**d**) boronized Ti6Al4V/HA composite, and (**e**) mineralized nodules of cells cultured on the different specimens for 14 days (*** *p* < 0.001).

Mineralized nodules are the mark of osteoblast mineralization and maturation, and the main morphological manifestation of osteoblasts to perform osteogenic function. Characterizing mineralized nodules of osteoblasts is one of the commonly used methods to study osteoblast mineralization [21]. Figure 5 shows alizarin red staining of cells on the surfaces of different groups of specimens, demonstrating the formation of mineralized nodules. 

As shown in Figure 5, many red nodules were scattered on the surface of Ti. For Ti6Al4V, more small red nodules were observed on its surface, which were scattered or gathered in a few areas. For boronized Ti6Al4V, there were less red mineralized nodules. For the boronized Ti6Al4V/HA composite, it had much larger and more red mineralized nodules on its surface than the other specimens. 

According to the above results, the cells on the boronized Ti6Al4V/HA composite had the most excellent mineralization ability, followed by Ti, Ti6Al4V, and boronized Ti6Al4V in the declining order. The formation of the large number of mineralized nodules on the surface of boronized Ti6Al4V/HA composite was mainly attributed to the presence of HA in the composite, which provided high concentrations of calcium and phosphorus ions in local environment, eventually promoting cell mineralization, continuous growth, and functional maturity of osteoblasts [22,23]. 

### 3.5. Postoperative Observation Results

Physical and mental conditions of all rabbits maintained good on the days after the surgeries. The diet, urine, and stool were normal, with satisfactory wound healing. There were no swelling, inflammation, infections, and other abnormal reactions. After separation of soft tissues and amputation of femurs, the implants were found to be completely embedded in the bone without loosening. 

### 3.6. Micro-CT Scanning Analysis

**Figure 6:**
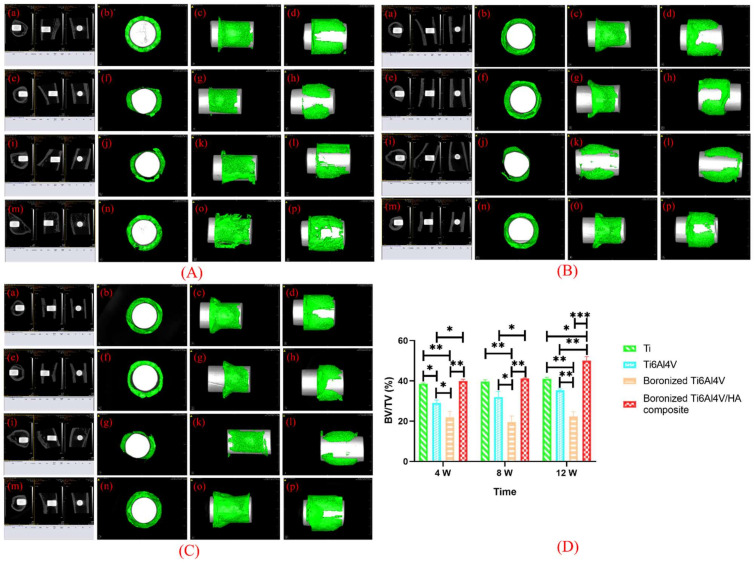
Characterization of implants and the surrounding bones by micro-CT scanning at the (**A**) 4th, (**B**) 8th, and (**C**) 12th weeks after implantation: (**a**–**d**) Ti, (**e**–**h**) Ti6Al4V, (**i**–**l**) boronized Ti6Al4V, and (**m**–**p**) boronized Ti6Al4V/HA composite. (**D**) Quantitative analysis of BV/TV by micro-CT (* *p* < 0.05, ** *p* < 0.01, and *** *p* < 0.001).

The femurs of the rabbits after implantation were scanned by micro-CT to quantitatively analyze the bone growth around the implants [24]. The bone growth on the cross-section, coronal plane, and sagittal plane of implants in each group at the 4th, 8th, and 12th weeks after operation and the three-dimensional reconstruction images are shown in Figure 6.

From Figure 6, it was observed that the amount of bone tissues on the surfaces of Ti and Ti6Al4V were relatively small. The amount of bone tissue on the surface of boronized Ti6Al4V was the least, featured by no new bone on its major surface. More and denser bone tissues were formed on the surface of the boronized Ti6Al4V/HA composite with good bone integration and high quantity of bone trabecula. 

Based on the analysis of the scanned images, the quantitative analysis of new bone formations around the implants in terms of BS, TV, BV, BS/BV, BS/TV, BV/TV, TB. N, Tb.Th, Tb.Sp, and BMD were performed. The results are shown in Table 1.

According to the analysis of the above parameters at the 4th, 8th, and 12th weeks, the variations of new bone formation with time were basically consistent. The growth of bone tissue around the boronized Ti6Al4V/HA composite was the best, followed by Ti, Ti6Al4V, and boronized Ti6Al4V. In addition, the values of BV/TV of the specimens at the 4th, 8th, and 12th weeks were determined, as shown in Figure 6D. According to the statistical analysis of BV/TV, at the 4th week, the boronized Ti6Al4V/HA composite had the largest BV/TV value, which was important for early bone formation. The BV/TV value of Ti was slightly lower than that of the boronized Ti6Al4V/HA composite (*p* > 0.05), indicating that Ti also had early osteogenesis. The corresponding values of Ti6Al4V and boronized Ti6Al4V decreased and there were statistical differences compared with the other groups (*p* < 0.05). At the 8th week, the BV/TV value increased slowly in all the groups except for boronized Ti6Al4V. At the 12th week, the BV/TV value of the boronized Ti6Al4V/HA composite increased significantly, with a statistical difference from the other groups (*p* < 0.05). The results indicated that the boronized Ti6Al4V/HA composite had the most obvious new bone formation. 

### 3.7. Toluidine Blue Staining and BIC Analysis

**Figure 7:**
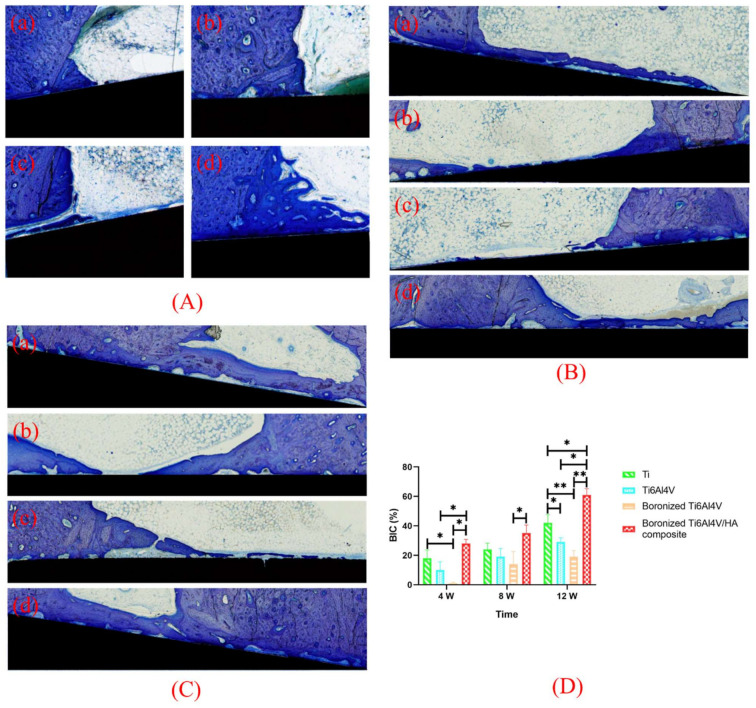
Toluidine blue staining of bone tissues around the different specimens at the (**A**) 4th, (**B**) 8th, and (**C**) 12th weeks after implantation: (**a**) Ti, (**b**) Ti6Al4V, (**c**) boronized Ti6Al4V, and (**d**) boronized Ti6Al4V/HA composite. (**D**) Quantitative analysis of BIC (* *p* < 0.05, ** *p* < 0.01).

The osteogenic ability of implants can be revealed by toluidine blue staining of bone tissues after implantation based on observation of new bones around the implants which presented different colors. The mineralized bone, osteoid, and implant are shown in light purple, pale blue, and black, respectively, to reflect formation characteristics of new bones at the interface between implant and bone [25]. The toluidine blue staining of bone tissues around the different specimens at the 4th, 8th, and 12th weeks after operation are shown in Figure 7. 

At the 4th week, a small amount of osteoid was formed outside the Ti implant, extending from the original cortical bone to the bone marrow cavity. An obvious inverted line was found between the newly formed osteoid and the original cortical bone, which appeared purple or blue. There was a small contact area between the osteoid and the implant. For Ti6Al4V, limited osteoid formation was observed around the implant whose boundary with the original cortical bone was not clear. For boronized Ti6Al4V, there was less osteoid on the surfaces of the original cortical bone and implant. For the boronized Ti6Al4V/HA composite, a large amount of pale blue osteoid was formed on its surface and there was a clear boundary between the newly formed bone and original bone. The surfaces of bone and the implant were contacted with no gap. At the 8th and 12th weeks, the amounts of osteoid on the surfaces of Ti and Ti6Al4V increased, but the thin layers of osteoid were not closely contacted with the implants due to the presence of obvious gaps. There were more osteoid and mineralized bone on the surface of the boronized Ti6Al4V/HA composite. They spread along the longitudinal axis of the implant with no obvious gap. 

Based on the quantitative analysis of toluidine blue staining, BIC, which was defined as the ratio of sum of contact length between trabecular bone and implant and contact length between cortical bone and implant to the perimeter of implant, was calculated to evaluate the osseointegration performance of the specimens. The BIC values of the different specimens at the 4th, 8th, and 12th weeks after operation are shown in Figure 7D.

According to toluidine blue staining and BIC calculation, at the 4th week, the bone formation in the boronized Ti6Al4V/HA composite was the most obvious, followed by Ti, Ti6Al4V, and boronized Ti6Al4V in the declining sequence. This means that the boronized Ti6Al4V/HA composite had faster and earlier bone formation, promoting bone integration. With the extension of implantation time to the 8th week, the number of osteoblasts gradually increased, which made the transformation of fiber tissue into fibrotic bone and osteoid on the implant surface along the longitudinal axis. As a result, the bone contact range increased. By 12 weeks, the new bone became gradually mineralized and dense. For the boronized Ti6Al4V/HA composite, there was a new bone band on the implant surface and the value of BIC increased significantly, which was statistically different from the other specimens (*p* < 0.05). Hence, it exhibited the excellent osseointegration. 

### 3.8. Push-Out Force Analysis

**Figure 8:**
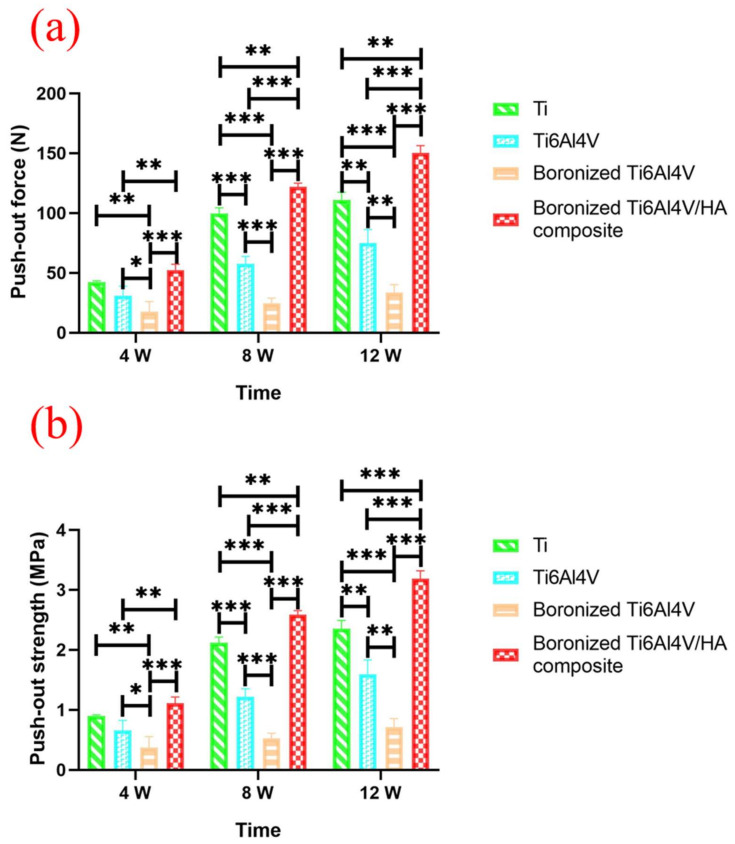
(**a**) Maximum push-out force and (**b**) push-out strength after surgery at the 4th, 8th, and 12th weeks (* *p* < 0.05, ** *p* < 0.01, and *** *p* < 0.001).

The push-out force tests were carried out for the implants at the 4th, 8th, and 12th weeks after operation and the force–displacement curves of each group were recorded. The values of maximum push-out force of each group of specimens at different time are summarized in Table 2 and their comparisons between the groups are shown in Figure 8.

As shown in Figure 8a, at the 4th week after operation, the boronized Ti6Al4V/HA composite presented the maximum push-out force. At the 8th week, the push-out force of each group increased significantly, especially in the boronized Ti6Al4V/HA composite, with the value increased by more than twice that at the 4th week. It was because of increased formation of new bone around the implant, which accelerated bone integration. The difference was significant statistically compared with the other groups (*p* < 0.01 or *p* < 0.001). The push out force of Ti, Ti6Al4V, and boronized Ti6Al4V declined in order, with statistical differences between any two of them (*p* < 0.05). At the 12th week, the push out force of all groups continued to rise steadily, and again, the boronized Ti6Al4V/HA composite showed the maximum push-out force, indicating that there was a larger contact area and better mechanical or chemical combination between the surrounding bone tissue and the implant, which supported bone integration. 

The push-out strength, defined as the ratio of maximum push-out force to the product of π, diameter, and height of implant, was also calculated, as shown in Table 2. The results of statistical analysis of the push-out strength (Figure 8b) at the 4th, 8th, and 12th weeks after operation were basically the same as those of Figure 8a. Except for the 12th week, the statistical difference between the boronized Ti6Al4V/HA composite and Ti became greater, namely from *p* < 0.01 to *p* < 0.001. 

The above results indicated that the boronized Ti6Al4V/HA composite had the most excellent osseointegration properties. It could be ascribed to many inspiring features of the composite. Firstly, the composite had a special surface composition, with involvement of both HA and boron. HA, the main component of the composite with excellent biological activity, played a key role in the process of bone conduction and new bone formation. In fact, HA has been widely used as implant coating in many previous studies to promote bone generation [26]. It was demonstrated that HA coating on the surface of metal implants could significantly increase bone conductivity with the contact area percentage of new bone formation to nearly 100% [27]. However, HA coating might also activate the macrophages around the implants, thus causing bone resorption. The HA coating had good osteogenic effects in the short and medium terms, but its long-term effect remains to be explored, due to coating decomposition, peeling, and falling off [28]. The boronized Ti6Al4V/HA composite prepared in this study could avoid the problem of HA falling off, maintaining a good bone conductivity potential to support bone matrix mineralization. The HA in the composite gradually dissolved in the body fluid and released high concentrations of calcium and phosphorus ions, which supported proliferation and differentiation of osteoblasts. Hence, this component was beneficial to biomineralization deposition for the new bone formation [29,30]. In addition, HA could be used as a carrier of proteins to induce ectopic osteogenesis and to accelerate the formation of new bone [31]. In addition to HA, the composite contained boron which is known as a trace element with good affinity for bone. In general, boron plays an important role in protecting joints, strengthening bone and accelerating fracture healing in human body [13]. Its use in implants can increase the number, width, and area of femur trabecular bone [32]. Moreover, it has the potential to inhibit the degradation of alveolar bone, tooth loosening and shedding, and to relieve the pain of disease [33]. Unlike common doping elements in implant materials, such as strontium, zinc, magnesium, lithium, and silver [34,35,36,37], boron has an estrogen-like effect and is a weak environmental estrogen substance, which may affect the estradiol level and estrogen receptor (ER) expression of ovariectoized rats [38]. This element can mediate the osteogenic differentiation of bone marrow mesenchymal stem cells through ERα, with higher activity of osteoblasts, promoted collagen synthesis, enhanced bone mineralization, and increased bone formation. In addition, boron can inhibit osteoclast differentiation and weaken bone resorption by regulating the OPG/RANKL/RANK signaling pathway [39]. The special composition of the composite also contribute to a good wettability with water contact angle of 44.90° [7]. This angle was much lower than those of other implants, especially Ti (83.5°) and Ti6Al4V (95.8°). After the implants contacted with blood during implantation, the biochemical pathway of coagulation system would be activated. As a result, there would be conversion of fibrinogen into fibrin, vasodilatation, and absorption of more blood protein molecules. The fibrin was then reintegrated into fibrin bundle in the blood clot. After stabilizing on the implant surface, the fibrin clot would form a mechanical lock with the surface (knock on effect) and the platelets were gathered and activated to produce growth factors, accelerating wound healing. This change could provide a scaffold for the migration of osteoblast precursor cells to the implant surface and promote the formation, growth, and functional activities of osteoblasts [40]. Secondly, the composite had a porous structure with the porosity of 11.25% [7], which was comparable to that of boronized Ti6Al4V (13.27%) but much higher than those of Ti and Ti6Al4V without evident pores. This feature could facilitate the growth of the newly formed bone tissue into micro-pores, producing close chimeras between bone tissue and implant surface. It would form strong mechanical locks, which increased the contact area of the implant surface and improved bone-implant interfacial strength [41]. Thirdly, the boronized Ti6Al4V/HA composite was prepared by pressing and sintering of Ti6Al4V, HA, and TiB_2_ powders. Its surface was rougher than commercial pure titanium and titanium alloy [7], resulting in a favorable micro-morphology. It could promote the adhesion, proliferation, differentiation, and mineralization of osteoblasts in vivo, providing sufficient adhesive sites for osteoblasts and extracellular matrix proteins, with enhanced expression of osteogenic genes and proteins and accelerated formation of new bone. The improved stability of the implant at the initial stage would contribute to the osseointegration, reducing osseointegration time and failure rate of initial implantation [42,43].

The future efforts will be spent on further improving the comprehensive performance of the composite from the perspectives of both physical and chemical modifications. For the former, optimizing the particle sizes of the raw materials, especially Ti6Al4V and TiB_2_ whose sizes were in the micro scale in this study, will be carried out to accelerate the preparation process of the composite with improved mechanical properties. For the latter, the ionic substitutions of HA, such as fluorination of HA, for preparing the composite will be considered to enhance the osteogenic response and bone regeneration of the material. 

## 4. Conclusions

The osteogenesis performance of the boronized Ti6Al4V/HA composite as dental implant was evaluated based on both cell experiments in vitro and animal experiments in vivo. The boronized Ti6Al4V/HA composite was prepared by microwave sintering of powders of Ti6Al4V, HA, and TiB_2_ in the gas of high-purity Ar at 1050 °C for 30 min. The adhesion, proliferation, ALP activity, and mineralization of MG-63 cells on the surfaces of specimens including Ti, Ti6Al4V, boronized Ti6Al4V, and boronized Ti6Al4V/HA composite were assessed in vitro. It was demonstrated that the boronized Ti6Al4V/HA composite exhibited the best inductive osteogenesis potential, in association with its bioactive HA component, porous structure, and relatively rough micro-morphology. In addition, the in vivo study in rabbits was performed based on the micro-CT analysis, histological and histomorphometric evaluation, and biomechanical testing, which verified that the boronized Ti6Al4V/HA composite had the highest new bone formation potential and osseointegration property after implantation for 4, 8, and 12 weeks. In particular, the bone volume fraction, bone implant contact, and maximum push-out force were 48.64%, 61%, and 150.3 ± 6.07 N at the 12th week. Because of these inspiring features, the boronized Ti6Al4V/HA composite is believed to be a very promising dental implant material.

## Figures and Tables

**Table 1:** Quantitative analysis of new bone formations around implants by micro-CT at the 4th, 8th, and 12th weeks after implantation.

Time	Group	BS (mm^2^)	TV (mm^3^)	BV (mm^3^)	BS/BV (1/mm)	BS/TV (1/mm)	BV/TV (%)	Tb.N (1/mm)	Tb.Th (mm)	Tb.Sp (mm)	BMD (g/cm^3^)
4 W	Ti	113.86	24.20	9.40	12.11	4.70	38.84	2.35	0.17	0.26	677.80
	Ti6Al4V	94.22	19.19	5.77	16.34	4.91	30.05	2.46	0.12	0.28	541.54
	Boronized Ti6Al4V	127.16	22.80	5.46	23.30	5.58	23.94	2.79	0.09	0.27	459.85
	Boronized Ti6Al4V/HA composite	145.64	24.14	9.48	15.36	6.03	39.27	3.02	0.13	0.20	634.29
8 W	Ti	113.93	23.06	9.29	12.26	4.94	40.30	2.47	0.16	0.24	634.35
	Ti6Al4V	112.08	19.98	6.78	16.54	5.61	33.92	2.81	0.12	0.24	756.00
	Boronized Ti6Al4V	103.90	27.68	4.81	21.58	3.75	17.39	1.88	0.09	0.44	315.28
	Boronized Ti6Al4V/HA composite	113.42	18.49	7.66	14.80	6.14	41.45	3.07	0.14	0.19	800.13
12 W	Ti	111.67	21.61	8.98	12.44	5.17	41.55	2.58	0.16	0.23	779.14
	Ti6Al4V	92.47	18.23	6.54	14.15	5.07	35.84	2.54	0.14	0.25	673.93
	Boronized Ti6Al4V	94.59	25.39	5.24	18.05	3.73	20.64	1.86	0.11	0.43	376.15
	Boronized Ti6Al4V/HA composite	138.93	24.14	11.74	11.83	5.76	48.64	2.88	0.17	0.18	934.36

**Table 2:** Maximum push-out force and push-out strength after surgery at the 4th, 8th, and 12th weeks.

Index	Group	4 W	8 W	12 W
Maximum push-out force (N)	Ti Group	42.4 ± 0.90	99.8 ± 4.58	111.0 ± 6.44
	Ti6Al4V Group	31.0 ± 7.92	57.6 ± 6.14	75.1 ± 11.15
	Boronized Ti6Al4V	17.5 ± 8.57	24.7 ± 4.17	33.6 ± 6.66
	Boronized Ti6Al4V/HA composite	52.4 ± 4.86	122.0 ± 3.16	150.3 ± 6.07
Push-out strength (MPa)	Ti Group	0.90 ± 0.02	2.12 ± 0.10	2.36 ± 0.14
	Ti6Al4V	0.66 ± 0.17	1.22 ± 0.13	1.59 ± 0.24
	Boronized Ti6Al4V	0.37 ± 0.18	0.52 ± 0.09	0.71 ± 0.14
	Boronized Ti6Al4V/HA composite	1.11 ± 0.10	2.59 ± 0.07	3.19 ± 0.13

## Data Availability

The data presented in this study are available on request from the corresponding author.

## References

[1] Duraccio D., Mussano F., Faga M.G. (2015). Biomaterials for dental implants: Current and future trends. J. Mater. Sci..

[2] Zhang C., Zhang T.J., Geng T.Y., Wang X.D., Lin K.L., Wang P.L. (2021). Dental implants loaded with bioactive agents promote osseointegration in osteoporosis: A review. Front. Bioeng. Biotech..

[3] Avila J.D., Stenberg K., Bose S., Bandyopadhyay A. (2021). Hydroxyapatite reinforced Ti6Al4V composites for load-bearing implants. Acta Biomater..

[4] Kumari R., Majumdar J.D. (2017). Studies on corrosion resistance and bio-activity of plasma spray deposited hydroxylapatite (HA) based TiO2, and ZrO2, dispersed composite coatings on titanium alloy (Ti-6Al-4V) and the same after post spray heat treatment. App. Surf. Sci..

[5] Alem S.A.A., Latifi R., Angizi S., Hassanaghaei F., Aghaahmadi M., Ghasali E., Rajabi M. (2020). Microwave sintering of ceramic reinforced metal matrix composites and their properties: A review. Mater. Manuf. Process..

[6] Rondanelli M., Faliva M.A., Peroni G., Infantino V., Gasparri C., Iannello G., Perna S., Riva A., Petrangolini G., Tartara A. (2020). Pivotal role of boron supplementation on bone health: A narrative review. J. Trace Elem. Med. Biol..

[7] Peng Q., Tang Z.G., Wang Y.H., Peng Z.W. (2019). Mechanical performance and in-vitro biological behaviors of boronized Ti6Al4V/HA composites synthesized by microwave sintering. Ceram. Int..

[8] Peng Q., Bin X., Xuxin H.Y., Wang Y.H., Peng Z.W., Tang Z.G. (2020). Facile fabrication of boronized Ti6Al4V/HA composites for loadbearing applications. J. Alloy. Compd..

[9] Chen S., Lee C.Y., Li R.W., Smith P.N., Qin Q.H. (2017). Modelling osteoblast adhesion on surface-engineered biomaterials: Optimisation of nanophase grain size. Comput. Method. Biomec. Biomed. Eng..

[10] Huang G., Yu H., Wang X., Ning B., Gao J., Shi Y., Zhu Y., Duan J. (2021). Highly porous and elastic aerogel based on ultralong hydroxyapatite nanowires for high-performance bone regeneration and neovascularization. J. Mater. Chem. B.

[11] Kukueva E.V., Putlyaev V.I., Tikhonov A.A., Safronova T.V. (2017). Octacalcium phosphate as a precursor for the fabrication of composite bioceramics. Inorgan. Mater..

[12] Barrak F.N., Li S., Muntane A.M., Jones J.R. (2020). Particle release from implantoplasty of dental implants and impact on cells. Int. J. Implant Dent..

[13] Dede E.C., Korkusuz P., Bilgiç E., Çetinkaya M.A., Korkusuz F. (2022). Boron nano-hydroxyapatite composite increases the bone regeneration of ovariectomized rabbit femurs. Biol. Trace Elem. Res..

[14] Shao Y., Qing X., Peng Y., Wang H., Shao Z., Zhang K. (2021). Enhancement of mechanical and biological performance on hydroxyapatite/silk fibroin scaffolds facilitated by microwave-assisted mineralization strategy. Colloids Surf. B Biointer..

[15] Mohammadtaheri M., Bozorg M., Yazdani A., Majid S. (2022). Fabrication of Ti-Al2O3-HA composites by spark plasma sintering and its properties for medical applications. J. Mater. Res..

[16] Taubenberger A.V., Woodruff M.A., Bai H., Muller D.J., Hutmacher D.W. (2010). The effect of unlocking RGD-motifs in collagen I on pre-osteoblast adhesion and differentiation. Biomaterials.

[17] Mariné O., María E.G.D., Antonio A.P.M., Amaury P.G., Christian G., Héctor F. (2017). Evaluation of the osteoblast behavior to PGA textile functionalized with RGD as a scaffold for bone regeneration. J. Nanomater..

[18] Li J., Ge L., Zhao Y., Zhai Y., Rao N., Yuan X., Yang J., Li J., Yu S. (2022). TGF-β2 and TGF-β1 differentially regulate the odontogenic and osteogenic differentiation of mesenchymal stem cells. Arch Oral Biol..

[19] Harjumäki R., Zhang X., Nugroho R.W.N., Farooq M., Sterberg M. (2020). AFM force spectroscopy reveals the role of integrins and their activation in cell-biomaterial interactions. ACS Appl. Bio Mater..

[20] Clark E.A., Brugge J.S. (1995). Integrins and signal transduction pathways: The road taken. Science.

[21] Assefa F., Kim J.A., Lim J., Nam S.H., Shin H.I., Park E.K. (2022). The neuropeptide spexin promotes the osteoblast differentiation of MC3T3-E1 cells via the MEK/ERK pathway and bone regeneration in a mouse calvarial defect model. Tissue Eng. Regen. Med..

[22] Li L.H., Kong Y.M., Kim H.W., Kim Y.W., Kim H.E., Heo S.J., Koak J.Y. (2004). Improved biological performance of Ti implants due to surface modification by micro-arc oxidation. Biomaterials.

[23] Han Y. (2021). High concentrations of calcium suppress osteogenic differentiation of human periodontal ligament stem cells invitro. J. Dent. Sci..

[24] Ramenzoni L.L., Bsch A., Proksch S., Attin T., Schmidlin P.R. (2020). Effect of high glucose levels and lipopolysaccharides induced inflammation on osteoblast mineralization over sandblasted/acid etched titanium surface. Clin. Implant Dent. Relat. Res..

[25] Ye J., Huang B., Gong P. (2021). Nerve growth factor-chondroitin sulfate/hydroxyapatite-coating composite implant induces early osseointegration and nerve regeneration of peri-implant tissues in Beagle dogs. J. Orthop. Surg. Res..

[26] Ritwik A., Saju K.K., Vengellur A., Saipriya P.P. (2022). Development of thin-film hydroxyapatite coatings with an intermediate shellac layer produced by dip-coating process on Ti6Al4V implant materials. J. Coat. Technol. Res..

[27] Rattan P.V., Sidhu T.S., Mittal M. (2012). An overview of hydroxyapatite coated titanium implants. AJEAT.

[28] Pinto T.S., Martins B.R., Ferreira M.R., Bezerra F. (2022). Nanohydroxyapatite-blasted bioactive surface drives shear-stressed endothelial cell growth and angiogenesis. BioMed Res. Inter..

[29] Tao Z.S., Wu X.J., Yang M., Xu H.G. (2019). Local administration with silymarin could increase osseointegration of hydroxyapatite-coated titanium implants in ovariectomized rats. J. Biomater. Appl..

[30] Wang H.J., Yang G.G., Zhang J.M., Li S.M., Muhammad B. (2022). Hydroxyapatite nanoparticles/polyimi.de-coated platinum electrodes for improved heat-insulating and heavy metal ion diffusion properties. JNC.

[31] Aval F.S., Arab M.R., Sargolzaei N., Noushadi F., Pour A.H. (2018). Efficacy of octacalcium phosphate and octacalcium phosphate/gelatin composite on the repair of critical-sized calvarial defects in rats. J. Dent..

[32] Abdelnour S.A., Abd El-Hack M.E., Swelum A.A., Perillo A., Losacco C. (2018). The vital roles of boron in animal health and production: A comprehensive review. J. Trace Elem. Med. Bio..

[33] Toker H., Ozdemir H., Balci Yuce H., Goze F. (2016). The effect of boron on alveolar bone loss in osteoporotic rats. J. Dent. Sci..

[34] Zhou C., Xu A.T., Wang D.D., Lin G.F., Liu T., He F.M. (2018). The effects of Sr-incorporated micro/nano rough titanium surface on rBMSC migration and osteogenic differentiation for rapid osteointegration. Biomater. Sci..

[35] He J., Feng W., Zhao B.H., Zhang W., Lin Z. (2018). In vivo effect of titanium implants with porous zinc-containing coatings prepared by plasma electrolytic oxidation method on osseointegration in rabbits. Int. J. Oral Maxillofac. Implant..

[36] Zhu Y., Zhang C.N., Gu Y.X., Shi J.Y., Mo J.J., Qian S.J., Qiao S.C., Lai H.C. (2019). The responses of human gingival fibroblasts to magnesium-doped titanium. J. Biomed. Mater. Res. A.

[37] Huang T.B., Li Y.Z., Yu K., Yu Z., Wang Y., Jiang Z.W., Wang H.M., Yang G.L. (2019). Effect of the Wnt signal-RANKL/OPG axis on the enhanced osteogenic integration of a lithium incorporated surface. Biomater. Sci..

[38] Song S., Gao P., Sun L., Kang D., Kongsted J., Poongavanam V., Zhan P., Liu X. (2021). Recent developments in the medicinal chemistry of single boron atom-containing compounds. Acta Pharm. Sin. B.

[39] Udagawa N., Koide M., Nakamura M., Nakamichi Y., Yamashita T., Uehara S., Kobayashi Y., Furuya Y., Yasuda H., Fukuda C. (2021). Osteoclast differentiation by RANKL and OPG signaling pathways. J. Bone Miner. Metab..

[40] Tavakol S., Nikpour M.R., Amani A., Soltani M., Rabiee S.M., Rezayat S.M., Chen P., Jahanshahi M. (2013). Bone regeneration based on nano-hydroxyapatite and hydroxyapatite/chitosan nanocomposites: An in vitro and in vivo comparative study. J. Nanoparti. Res..

[41] Causey G.C., Picha G.J., Price J., Pelletier M.H., Wang T., Walsh W.R. (2021). In-Vivo response to a novel pillared surface morphology for osseointegration in an ovine model. J. Mech. Behav. Biomed. Mater..

[42] Bruyn D.H., Véronique C., Doornewaard R., Jacobsson M., Cosyn J., Jacquet W., Vervaeke S. (2017). Implant surface roughness and patient factors on long-term peri-implant bone loss. Periodontology.

[43] Shanbhag S., Shanbhag V., Stavropoulos A. (2015). Genomic analyses of early peri-implant bone healing in humans: A systematic review. Inter. J. Imp. Dent..

